# Sexual and Reproductive Health and Rights and Global Development

**DOI:** 10.1111/sifp.70016

**Published:** 2025-05-22

**Authors:** Gilda Sedgh, Susheela Singh, Irum Taqi, Jonathan Wittenberg

## Abstract

The ICPD Programme of Action and the Sustainable Development Goals both underscore the essential role of sexual and reproductive health and reproductive rights in development. Despite significant progress on many aspects of sexual and reproductive health and rights (SRHR), challenges remain, and they are exacerbated by rising anti‐rights movements in many countries. At a time when SRHR is under threat, it is important to surface evidence that speaks to its critical role in development and its inextricable connections to multiple global goals. In this commentary, we argue that investing in SRHR is strategic because it yields substantial benefits to individuals, economies, societies, the environment, and peace and security, and thus contributes to progress on related goals. We encourage SRHR advocates to leverage the broad array of arguments to bolster decision‐makers’ and other stakeholders’ support of SRHR, alongside the well‐established arguments grounded in cost‐effectiveness, and returns on health and human rights. With the world falling short of achieving the Sustainable Development Goals and conservative forces threatening to undo the progress that has been made, urgent and collective action on multiple fronts is needed. By recognizing that many development priorities are interconnected, we can accelerate progress through cross‐movement advocacy and mobilization.

## INTRODUCTION

The ICPD Programme of Action (PoA) of 1994 (United Nations 1994) accelerated momentum toward achieving reproductive health and rights for all. Some 20 years later, the centrality of sexual and reproductive health (SRH) to development was affirmed in the Sustainable Development Goals (SDGs) (UN General Assembly, Seventieth Session 2015), which included two relevant targets: “universal access to sexual and reproductive health‐care services” and “universal access to sexual and reproductive health and reproductive rights.” In 2018, the Guttmacher–*Lancet* Commission (Starrs et al. 2018) put forth a comprehensive definition of sexual and reproductive health and rights (SRHR) that expanded the vision set forth by the ICPD PoA and the Beijing Declaration by fully integrating sexual rights as an essential dimension of SRHR.

Sexual and reproductive rights are recognized as human rights (United Nations 1994; Shalev 2000; UNFPA, Danish Institute for Human Rights, and Office of the United Nations High Commissioner for Human Rights 2014) and access to quality SRH services is essential for fulfilling these rights. There is also a robust body of evidence showing that investing in SRH services yields substantial returns in the form of health outcomes and cost savings: SRH care provides significant benefits for the health of women, their partners, infants and children, and cost savings to health systems (Sully et al. 2020). Moreover, maternal health care services, which are integral components of comprehensive SRHR, form the backbone of primary health care (WHO 2004), and strengthening the delivery of these services strengthens health systems and their ability to provide a range of essential services (Population Action International 2023; USAID 2024).

Unfortunately, despite a strong and growing body of evidence supporting these health, cost, and rights‐based arguments, they are often not enough to motivate policymakers and donors to prioritize SRHR. Exacerbating this problem in recent years is the fact that well‐funded anti‐rights movements (Gilby and Koivusalo 2024) have made it even harder to enact policies and secure financing to advance SRHR, or even to protect existing programs and policies. The starkest manifestation of this new political landscape is the recent withdrawal of U.S. foreign assistance for global SRHR (The White House 2025). Other significant challenges to progress include persistently inadequate resources, an unwillingness to address issues related to sexuality openly, increasingly widespread dissemination of dis‐ and misinformation, and weak political commitment of some low‐ and middle‐income country (LMIC) governments to SRHR.

However, the arguments for investing in SRHR are much more far‐reaching than those centered on health, cost savings, and rights. The realization of SRHR also catalyzes progress on other development priorities, including gender equality, economic growth, climate change resilience, and peace and security. This moment calls for reinvigorating and expanding the base of support for SRHR, including by marshaling the considerable evidence on the broad benefits of achieving global SRHR goals. In this commentary, we briefly highlight the progress that has been made so far in advancing SRHR and then make an evidence‐based case for investing in SRHR as a means of achieving progress on multiple development priorities.

## PROGRESS ON SRHR

There have been many advancements in SRHR since the 1994 ICPD. Notably, the expansion of family planning programs has contributed to a 22 percent decline between 1990–1994 and 2015–2019 in unintended pregnancies among women wishing to avoid pregnancy globally, and 30 percent in sub‐Saharan Africa (Bearak et al. 2023). The global maternal mortality ratio (MMR) fell by 34 percent between 2000 and 2020 (WHO, UNICEF, and UNFPA 2023). Global efforts to combat HIV have led to a 60 percent reduction in new infections since 1995 (HIV.gov 2025). And the universal health coverage subindex for reproductive, maternal, newborn, and child health rose by 11 percent between 2000 and 2021, reflecting improvements in service availability and quality (WHO 2025).

But major challenges persist. Despite the long‐term downward trend, detailed estimates indicate that the MMR stalled after 2016 (United Nations 2024b). Disparities in the MMR also persisted, with sub‐Saharan Africa and Southern Asia accounting for 87 percent of all maternal deaths globally. Adolescent fertility remains high, with nearly one in three young women giving birth before they are 20 years old, and rates are particularly high in sub‐Saharan Africa and Latin America (UNFPA 2022). Gender‐based violence (GBV) remains a major global issue and one in three women worldwide experiences intimate partner or nonpartner sexual violence in her lifetime, by most recent estimates (WHO 2021). Comprehensive sexuality education remains inconsistently implemented (UNESCO 2021).

Reproductive cancers, largely overlooked in the ICPD PoA, have also become a pressing issue, with rising breast cancer cases in multiple regions (Huang et al. 2021). Among women diagnosed with breast and cervical cancer—which are among the most common types of cancer among women worldwide—the risk of death is substantially higher in LMICs than in high‐income countries (HICs) (Ginsburg et al. 2017).

Care for infertility and subfertility is another aspect of SRHR that is often overlooked, and while assisted reproductive technologies have become more widely available in many HICs, fewer than 1.5 percent of people have access to assisted reproduction in Africa (Ombelet and Onofre 2019).

Comprehensive SRHR includes other components that are inadequately measured or monitored, such as those related to the sexual health and well‐being of people of all ages (Vasconcelos et al. 2024).

## SRHR AND OTHER DEVELOPMENT PRIORITIES

### SRHR and Gender Equality

SRHR contributes to and is intrinsically linked to gender equality, as reflected in major documents such as the SDGs Agenda Resolution and the Platform for Action of the 1995 United Nations World Conference on Women in Beijing (UN Women 1995). The Beijing platform articulates that “in most countries, the neglect of women's reproductive rights severely limits their opportunities in public and private life, including opportunities for education and economic and political empowerment.” The SDG subgoal of universal access to sexual and reproductive health and reproductive rights falls under the main gender equality goal because it is seen as a means to “achieve gender equality and empower all women and girls” (UN General Assembly, Seventieth Session 2015).

However, progress toward achieving gender equality has slowed globally, and it has been uneven (UN Women and DESA 2024). The World Economic Forum estimates that, at the current rate of progress, it will take 134 years to achieve global gender equality (World Economic Forum 2024). Low‐income countries, particularly in sub‐Saharan Africa, South Asia, and the Middle East and North Africa, face the most sizeable gender gaps.

SRHR as a foundation of gender equality starts with comprehensive sexuality education and access to adolescent friendly services. Equipping girls and young women with knowledge and the tools to manage their sexual and reproductive health enables them to make informed decisions on central aspects of their lives, including their relationships and their broader goals (Mirzaii Najmabadi and Sharifi 2018; Parida, Gajjala, and Giri 2021). Quality sexuality education programs for all young people, including boys, also promote gender‐equitable norms and values and help dismantle harmful stereotypes and power imbalances (Parida, Gajjala, and Giri 2021; UNESCO et al. 2018), and they help women build their decision‐making skills and self‐efficacy, which help them to be more equal partners in a relationship or marriage. At present, a lack of access to sexuality education as well as contraceptive services contributes to 21 million adolescents aged 15–19 in LMICs getting pregnant each year (Sully et al. 2020).

Access to contraception throughout one's reproductive years is another core element of SRHR, and studies have demonstrated positive associations between women's ability to delay, space, and limit births and their social and economic empowerment (Upadhyay et al. 2014). One of the most straightforward pathways is through education: when women can delay childbearing, they can stay in school longer and reap the social and economic benefits that an education confers, and this fosters a more gender‐equitable distribution of resources and opportunities. It also reduces women's financial dependence on their partners.

Comprehensive SRHR also includes freedom from GBV (Starrs et al. 2018). SRHR programs that tackle GBV ease the way for women—as well as transgender men and gender nonconforming people—to freely pursue educational and economic opportunities; they also challenge harmful and discriminatory gender stereotypes, and by breaking the cycle of violence they yield benefits that can last for generations.

### SRHR and Economic Growth

There is ample evidence of the economic gains associated with the realization of SRHR and of the threats to productivity and economic growth that follow from poor SRHR outcomes.

Enabling women to control their fertility, whether through contraception or abortion, allows them to enter into and advance in the formal labor force and enables them to reap economic benefits for themselves and their families, and even to bolster national economies (UNFPA 2024; Family Planning Impact Consortium 2023). This is evident even in resource‐poor settings, indicating that countries need not wait for broader economic development to achieve these returns (Family Planning Impact Consortium 2023). Youth‐friendly services, including sexuality education and protections against child marriage and early pregnancy, set the stage for women to contribute to societies and economies.

When women have reproductive autonomy and the means to implement their childbearing preferences, they generally have smaller families. At the societal or macro level, this decline in family size leads to a period of opportunity referred to as the “demographic dividend,” during which the ratio of working‐age adults to dependent children is high (Canning and Schultz 2012). Under the right conditions, such as those where governments invest in their human capital and adopt policies that create diverse employment opportunities for men and women, this window of opportunity bolsters economies in lasting ways (Navaneetham and Dharmalingam 2012; Cardona et al. 2020).

Just as access to SRH quality services can enhance economic productivity and growth and the financial stability of families and individuals, the lack of access to care, resulting in poor SRH outcomes, can hinder it. A useful summary indicator of this impact is the number of productive years of life lost due to ill‐health and death—referred to as disability‐adjusted life years (DALYs)—resulting from poor SRH. Several components of SRH contribute significantly to DALYs, and the majority of these DALYs could be prevented with access to SRH services. In the absence of adequate care, these health outcomes have large impacts on people's ability to participate effectively in the workforce. The ripple effect on families can also be severe, as surviving children may suffer from neglect, reduced educational opportunities, and poor health, perpetuating a cycle of poverty.

Poor maternal health outcomes and maternal deaths are responsible for 12 million years of productive life lost annually, by the most recent estimates (IHME n.d.). Reproductive cancers also compromise the health and productivity of millions of women each year and cervical cancer is one of the leading causes of cancer death among women in LMICs (Bray et al. 2018; Gossa and Fetters 2020), and it accounts for the loss of nine million DALYs annually around the globe (Global Burden of Disease 2019 Cancer Collaboration et al. 2022). Breast cancer, which also disproportionately affects women in LMICs, is responsible for 20.3 million DALYs annually. Finally, HIV/AIDS accounts for 47.6 million DALYs annually, with 25–54‐year‐old men and women, and particularly women aged 15–24 in sub‐Saharan Africa, facing the heaviest burden of illness and death (Tian et al. 2023).

Perhaps less well‐known are the economic consequences of gender‐based violence, which has been shown to compromise women's ability to contribute fully to the labor force. The International Monetary Fund estimated that every one percentage point increase in the share of women subject to GBV reduces economic activity, measured using proxies for GDP, by as much as 8 percent in the region of sub‐Saharan Africa (Ouedraogo and Stenzel 2021). The global cost of intimate partner violence has been estimated at 5 percent of worldwide GDP and nearly 15 percent of GDP in sub‐Saharan Africa (Yount et al. 2022).

### SRHR and Climate Change Resilience

The health of a country's population affects its ability to cope with and adapt to climate change, and investments in population health can reduce the impacts of climate change (Women Deliver 2021). Emerging evidence demonstrates linkages between women's access to and uptake of SRH services and their engagement in climate action (Dazé 2021; MSI, n.d.). Women are often responsible for managing the use of natural resources such as water and land, and when they have access to SRH services they are more likely to stay in school, engage as wage earners, and participate in informed decision‐making at the community level, which leads to more sustainable use of natural resources.

Investing in SRH care can also mitigate the negative health consequences of climate change. Climate events are associated with negative maternal and newborn health outcomes and increased incidence of gender‐based violence, HIV, and early and forced marriages (Arunda et al. 2024); strengthening SRH programs can attenuate these negative effects of climate change.

### SRHR and Peace and Security

SRHR benefits peace and security through its contributions to poverty reduction and economic well‐being. Societies with high rates of unwanted fertility typically have large adolescent and young adult populations at risk of unemployment and poverty. Youth bulges are linked to an increased risk of conflict when opportunities for young people to finish schooling, enter the labor market, and participate in governance are limited (Urdal 2011). When fertility rates align with women's intentions and investments in each child rise, societies can sustain political stability as well as economic prosperity (Hailu Demeke 2022; Azeng and Yogo 2013). Fulfilling women's desires for smaller families also creates more gender‐equitable opportunities to participate in political spheres, and it has been shown that peace processes and political negotiations that meaningfully include women are more likely to lead to successful and sustainable outcomes than those that do not (Eckert et al. 2017; Adjei 2019).

### SRHR and Nutrition

SRH services improve population nutrition and confer related benefits in several key ways. Contraception enables women to space their pregnancies, and in low‐resource settings healthy spacing of births gives mothers the time they need to recover nutritionally between pregnancies and is associated with lower rates of infant death (Molitoris, Barclay, and Kolk 2019). Adolescent pregnancies are also associated with higher risks of wasting and being underweight in children in LMICs, a link that has been attributed in part to maternal nutritional status (Welch et al. 2024), and programs that help young women prevent unwanted pregnancies and births help to reduce these risks. People living with HIV/AIDS often face challenges related to malnutrition due to the disease and its treatment, and SRH services that provide treatment for and prevention of HIV/AIDS improve nutritional outcomes (Cheah 2025). And perhaps most broadly, SRH services, which make it possible for women and girls to pursue their education and earn better incomes, position them to make informed decisions about food and nutrition for themselves and their families.

## CONCLUSIONS

The world is undeniably falling short of achieving the SDGs in social and economic development, gender equality, climate action, health, and SRHR. At the same time, we are facing myriad challenges to progress, including the increasing divisiveness being instigated by national and international actors and the growing strength of far‐right movements in a number of countries across the Global North and South. In response, we need urgent and collective action. By recognizing that many development priorities are crucial and deeply interconnected, we can accelerate progress through collaboration and mobilization across sectors.

While this review addresses the broad benefits that societies and economies can realize through investments in SRHR, it is important to emphasize that these relationships are bidirectional: improved social and economic conditions also pave the way toward progress on SRHR. For example, economic growth, political stability, and strong healthcare infrastructures make it easier to deliver SRH services, and equitable gender norms can foster the realization of reproductive rights and access to care. As the Commission on Population and Development observed in 2024, the ICPD PoA and the Sustainable Development Agenda “are mutually reinforcing” (Commission on Population and Development 2024). Indeed, advancing these agendas can foster virtuous cycles.

The evidence shows that a comprehensive approach to SRHR, as set out by the Guttmacher–*Lancet* Commission (Starrs et al. 2018), is essential to achieving the SDGs and realizing the full potential of our societies and economies. Governments can realize synergies by integrating a package of essential SRH services into universal health coverage and establishing sustainable mechanisms for financing these services. Enabling people to take full advantage of these services also requires implementing laws, policies, and programs that address barriers to the achievement of SRHR. States should also ensure that regional protocols support and ensure these rights.

As the global landscape of development assistance shrinks with political shifts to the right in some HICs, including the United States, historically the world's largest provider of development assistance, the need for diversification of funding sources has never been more essential.

Innovative approaches to international financing for SRHR, stronger mechanisms for domestic healthcare financing, and the integration of SRHR into other development policies and programs can fuel progress in all sectors. And while the available evidence demonstrates that SRHR and other development outcomes are inseparable, more research on these relationships will strengthen the case for targeted policy responses.

The economic, social, health, and environmental arguments for SRHR are clear. It is equally clear that these arguments are not alternatives to a rights‐based case but rather complementary to it. The opportunity to fully realize one's SRHR is not a privilege; it is a human right, essential to human dignity. The fulfillment of these rights is not just a strategic choice; it is also a moral and human obligation. This rights‐based argument must remain at the core of the case for investing in SRHR, even as we build on the growing understanding of the multiple ways in which progress on development priorities is inseparable from progress on SRHR and draw on complementary arguments supported by the evidence. The outcome document of the 2024 Summit for the Future reaffirms that economic development, peace and security, environmental sustainability, and human rights are interlinked and mutually reinforcing (United Nations 2024a). It recognizes that “sustained, inclusive and equitable economic growth, and sustainable development can only be realized when all women… and girls have their full human rights respected, protected and fulfilled,” and it names universal access to SRHR as one of the means toward these ends.

As we look beyond the first three decades of the ICPD era, and even beyond the SDG era, we can accelerate progress toward the full realization of SRHR and greater social and economic development by making use of the full range of evidence‐based arguments on how progress in each of these areas reinforces progress on the others. By working together across sectors through existing and new partnerships, we can accelerate progress on all of these fronts.
